# Individual and contextual factors associated to the self-perception of oral health in Brazilian adults

**DOI:** 10.11606/S1518-8787.2018052000361

**Published:** 2018-03-14

**Authors:** Janmille Valdivino da Silva, Angelo Giuseppe Roncalli da Costa Oliveira

**Affiliations:** I Universidade Federal do Rio Grande do Norte. Faculdade de Odontologia. Programa de Pós-Graduação em Saúde Coletiva. Natal, RN, Brasil; II Universidade Federal do Rio Grande do Norte. Faculdade de Odontologia. Departamento de Saúde Coletiva. Natal, RN, Brasil

**Keywords:** Adult, Diagnostic Self Evaluation, Oral Health, Socioeconomic Factors, Health Surveys

## Abstract

**OBJECTIVE:**

To analyze how individual characteristics and the social context, together, are associated with self-perception of the oral health.

**METHODS:**

A multilevel cross-sectional study with data from the Brazilian National Health Survey 2013, the United Nations Development Program, and the National Registry of Health Establishments. The explanatory variables for the “oral health perception” outcome were grouped, according to the study framework, into biological characteristics (sex, color, age), proximal social determinants (literacy, household crowding, and socioeconomic stratification), and distal (years of schooling expectancy at age 18, GINI, Human Development Index, and *per capita* income). The described analysis was performed, along with bivariate Poisson analysis and multilevel Poisson analysis for the construction of the explanatory model of oral health perception. All analyzes considered the sample weights.

**RESULTS:**

Both the biological characteristics and the proximal and distal social determinants were associated with the perception of oral health in the bivariate analysis. A higher prevalence of bad oral health was associated to lower years of schooling expectancy (PR = 1.31), lower *per capita* income (PR = 1.45), higher income concentration (PR = 1.41), and worse human development (PR = 1.45). Inversely, oral health services in both primary and secondary care were negatively associated with oral health perception. All the biological and individual social characteristics, except reading and writing, made up the final explanatory model along with the distal social determinants of the Human Development Index and coverage of basic care in the multilevel analysis.

**CONCLUSIONS:**

Biological factors, individual and contextual social determinants were associate synergistically with the population’s perception of oral health. It is necessary to improve individual living conditions and the implementation of public social policies to improve the oral health of the population.

## INTRODUCTION

Individual self-perception of health has been increasing its importance as a parameter for health status assessment of the population. As a measure, it can be considered a strong health predictor due to its relationship with clinical conditions and other indicators of both morbidity and mortality^1^.

In some countries with huge populations (as in Brazil), performing epidemiological population-based studies is usually very expensive and requires a great amount of both human and technological resources. In addition, clinical examinations are needed in some studies, increasing the resources needed and hence making them unfeasible^2^ ^,^ ^3^. The self-perception of health associated with treatment needs, as well as the degree of satisfaction with health status, have been used more frequently in health surveys.

Therefore, some population-based studies such as the National Health Research (PNS), performed between 2013 and 2014, have used the self-perception of the Brazilian population to obtain information about morbidity, risk factors, and healthy lifestyles^4^.

It is important to highlight the subjectivity beyond the self-assessment of health because its measure is a result of a complex web of factors, which includes individual characteristics, personal experiences, and also the environment where these subjects are living^5^ ^,^ ^6^. Nogueira^7^ indicates that the production and distribution of health is associated a relationship that man keeps with the social world. Mansuyr et al.^5^ observing the relationship between social environment and health in 45 countries showed an association between socioeconomic inequality and social capital with a self-perception of health, strengthening the importance of the theory of social health determination.

Regarding oral health, this context is no different. Oral health self-perception is also associated with individual factors and usually reveals an association with social factors^8–10^. In Brazil, the poor perception of oral health has been associated with low income and schooling, deteriorated housing conditions, inequality of income distribution, among other social factors^6^ ^,^ ^11^.

As a perspective to analyze the oral health conditions of populations, studies on social health determinants have been considered relevant for the identification of future strategies focused on oral health promotion. Considering that oral health promotion refers to actions on the social health determinants, aimed at favorably impacting the quality of life of individuals, according to the World Health Organization.

Despite this, nowadays, few studies^11–14^ have analyzed the association of both individual and contextual characteristics, together, with the oral health perception. Thus, this study aimed to analyze how individual conditions in combination with the social context where they are living are associated with the oral health perception in Brazilian adult population.

## METHODS

Study of the association between a dependent variable (oral health self-perception) and several independent variables, using a multilevel regression model. Secondary data were gathered from the PNS 2013 database, the main source of our individual data. This database was linked to two others, both with aggregated data from the state (the Brazilian federation unit) level: (a) the National Census, performed by the Brazilian Institute of Geography and Statistics (IBGE) with data compiled by the Brazilian agency of the United Nations Development Program (UNDP), which has created several indicators such as the Human Development Index (HDI) among others, and (b) the National Health Facilities Register (CNES), which contains information concerning public and private health services for the whole country.

There are several models for the social health determinants. Most of them have a similar structure, usually including different levels for explaining the social determination. We decided on the model proposed by Dalgren and Whitehead, based on layers representing specific levels of determination. In the first level, determinants are related to individual characteristics (age, sex, and genetic factors). In a second layer, above the previous one, there are the individual lifestyle factors and another layer representing the social and community networks. In the outermost layer, there are the general socioeconomic, cultural, and environmental conditions^15^.

The theoretical model was built taking this model as a reference and including recent findings regarding the factors associated to oral health perception (Figure). As proximal factors, we included the variables sex and skin color and healthy eating habits, representing lifestyle factors. For the socioeconomic characteristics, the model has included classical factors related to socioeconomic status (an indicator based on possession of goods) and formal education. All these variables were gathered from the PNS 2013 (Table 1).

**Figure:**
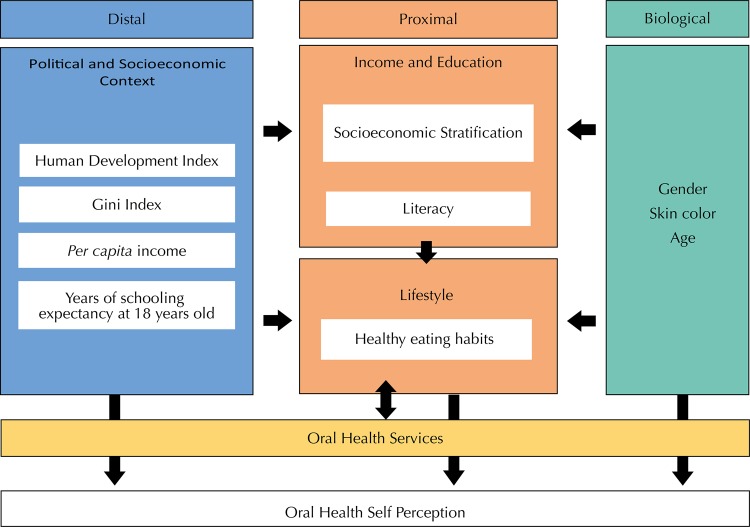
Framework of the study. Brazil, 2013.

**Table 1:** Individual and contextual variables. General description and adaptation strategies of the analysis model. Brazil, 2013.

Variable			Source	Description	Original categories (new categories)
Dependent		Oral Health Self-Perception	National Health Research 2013 (PNS, 2013)		Very good (Good) Good (Good) Not good, not bad (Bad) Bad (Bad) Very bad (Bad)
Independent (Individual)	Demographic	Gender	National Health Research 2013 (PNS, 2013)	Gender of interviewee.	Male and Female, no modification
		Race (skin color)	National Health Research 2013 (PNS, 2013)	Self-reported kin color of interviewee.	White (white) Black (non-white) Asian (non-white) Mixed color (non-white) Indian (non-white)
		Age	National Health Research 2013 (PNS, 2013)	Age, in years, at the moment of the interview.	Years, from 18 to 99, categorized to: 18–24 25–40 41–59 60 and older
	Socioeconomic	Literacy	National Health Research 2013 (PNS, 2013)	Answer to the question “Can you read and write?”	Yes or No, no modification
		Socioeconomic stratification	National Health Research 2013 (PNS, 2013)	The “Brazil Criterion of Economic Classification”^16^. It is based on possession of goods, such as a car, microcomputer, refrigerator, TV, washing machine, DVD player, microwave, motorcycle and mobile phone. The presence of bathroom and housekeeper were also recorded, and the householder’s educational status was also included. Each item has different weights. The variable represents the families’ purchasing power.	A numeric value, ranging from 0 to 46. Classes were defined as follows: A1 – 42 to 46 (A or B) A2 – 35 to 41 (A or B) B1 – 29 to 34 (A or B) B2 – 23 to 38 (A or B) C1 – 18 to 22 (C) C2 – 14 to 17 (C) D – 8 to 13 (D or E) E – 0 to 7 (D or E)
	Lifestyle	Healthy eating habits	National Health Research 2013 (PNS, 2013)	The variables consume salad, vegetables, fruit juice, soft drinks, and candies were included in a factorial analysis, creating two new factors. The factor containing salad, vegetables, fruit juice was denomination “health eating habits”.	A numeric value, categorized from median as “Yes” or “No”
Independent (Context)	Socioeconomic	Years of schooling expectancy at 18 years old	National Census, performed by the Brazilian Institute of Geography and Statistics (IBGE, from the Portuguese acronym)	Average of years of schooling that a child should complete before 18 years old.	Numeric value, categorized from the median in: 9.4 years and more Up to 9.4 years
		*Per capita* income	National Census, performed by the Brazilian Institute of Geography and Statistics (IBGE, from the Portuguese acronym)	The sum of the income of all household members, divided by the number of residents.	Numeric value, categorized from the median in: BR R$671 or more Up to BR R$670
		Gini Index	National Census, performed by the Brazilian Institute of Geography and Statistics (IBGE, from the Portuguese acronym)	An index to measure the inequality in the distribution of *per capita* income. Ranging from 0, where there is no inequality, to 1 when all income is concentrated in one single individual.	Numeric value, categorized from the median in: Up to 0.600 0.601 and more
		Human Development Index (HDI)	Brazilian agency of the United Nations Development Program (UNDP)	Geometric average of three other indices related to education, income, and longevity, with equal weights.	Numeric value, categorized from the median in: 0.709 and more Up to 0.708
	Oral Health Services	Oral Health Primary Care	National Health Facilities Register (CNES from the Portuguese acronym)	Number of oral health teams in primary care services, divided by the population in the same year.	Numeric value, categorized from the median in: 14.7 and more Up to 14.6

The PNS 2013 was a national household-based survey that aimed to characterize the health status and lifestyles of the Brazilian population, as well as aspects related to health care^16^. This survey was based on cluster sampling, using three stages: census tracks (Primary Sample Unity – PSU); households (Secondary Units) and an adult resident (Tertiary Unit) selected from a list made at the time of the interview. Due to this, the survey has different sample weights for the PSU, for the households, and for the selected resident. Thus, data from more than 60 thousand adults were used, which were selected for the interviews, and the analysis considered the complex sample design.

In the distal level, we included variables related to socioeconomic context plausibly associated with oral health status. Therefore, we included the average of *per capita* income, the Gini index, representing income inequality, and the years of schooling expectancy at 18 years old, meaning the educational context. We also included the Human Development Index (HDI), which is a combined measure of income, education, and longevity. We included the presence of oral health teams in primary care services, which means the population coverage and hence the possibility to obtain access to oral health services (Figure).

Multilevel modeling was used to assess both individual and contextual determinants. In multilevel analysis, the contextual levels can be considered as social aggregates, as they have a significant effect on their members. Therefore, individuals are usually considered as the first (lowest) level and the communities (neighborhood, cities or states, for example) where they are living are the second (upper) level^17^.

All variables were analyzed to verify the presence of missing data and outliers. From the PNS 2013, we extracted 64,308 records, corresponding to all the individuals interviewed, of 18 years and older. The PSN 2013 sample was representative for a Brazilian adult population aggregated at the State level. There were no missing data for age, skin color, reading and writing ability, socioeconomic stratification (Brazil Criterion^18^), healthy eating habits, and all contextual variables. Oral health perception had 6.4% of missing data and sex had only three missing cases. According to Hair Jr et al.^19^, missing data below 10% can be ignored, as long as they are missed at random. The missing data were Missing Completely at Random (MCAR). As we used complete data and also considering the sample size, the significance of associations was not affected. The final model counted a sample of 60,199 individuals. At the contextual level, all data were available.

A descriptive analysis was performed, aiming to identify cut-off points or other criteria to categorize the variables. As the final variables were created, association tests such as Rao Scott chi-square test were performed between the outcome (oral health self-perception) and all independent variables, selecting those with p-value ≤ 0.2, to be included in the multiple regression. Poisson regression analysis with robust variance was initially performed at an individual level to estimate the Prevalence Ratio with a respective 95% confidence interval. A Poisson Multilevel Regression Model was performed, including variables from different levels. The modeling was initiated by a null model to verify random effects, and variables from each dimension were included (demographic, socioeconomic, lifestyle etc.). The term of interaction between the socioeconomic contextual and individual variables was created to analyze the cross-level interaction.

## RESULTS

Regarding the general characteristics of the sample, 52.9% were women, 52.5% self-reported as non-white, 66.0% were aged between 25 to 59 years, 91.5% were literate, 39.6% were “class C” according to the “Brazil criterion” and 52.7% were individuals with healthy eating habits. The years of schooling expectancy was 9.6 years and the average *per capita* income was R$729.00. Gini index presented an average of 0.577, HDI was 0.711. The population rate of oral health teams in primary care was 14.39 and 0.53 per 100,000 inhabitants. In general, 67.4% of interviewees related their oral health as being good or very good (Table 2).

**Table 2:** Descriptive analysis of the variables according to the levels. Brazil, 2013.

Variable	%	95%CI
1st level (individual)		
Oral Health Self-Perception		
Good or very good	67.4	66.7–68.1
Regular to very bad	32.6	31.9–33.3
Sex		
Male	47.1	46.4–47.9
Female	52.9	52.1–53.6
Skin color		
White	47.5	46.7–48.3
Black or Mixed	52.5	51.7–53.3
Age (years)		
18 to 24	15.9	15.4–16.5
25 to 40	33.6	32.9–34.3
41 to 59	32.4	31.7–33.1
60 and older	18.0	17.5–18.7
Literate		
Yes	91.5	91.1–91.9
No	8.5	8.1–8.9
Socioeconomic stratification		
A or B	25.9	25.3–26.9
C	39.6	38.9–40.2
D or E	34.5	34.0–35.1
Healthy eating habits		
Yes	52.7	51.9–53.6
No	47.3	46.4–48.1

2nd level (state)		

Years of schooling expectancy at 18 years old		
9.4 and more	60.7	60.1–61.2
Up to 9.4	39.3	38.8–39.9
*Per capita* income (BR R$)		
671 and more	65.9	65.4–66.5
Up to 670	34.1	33.5–34.6
Gini Index		
Up to 0.600	68.0	67.4–68.5
0.601 and more	32.0	31.5–32.6
Human Development Index (HDI)		
0.709 and more	65.9	65.4–66.5
Up to 0.708	34.1	33.5–34.6
Oral Health Primary Care		
14.7 and more	49.5	48.9–50.1
Up to 14.6	50.5	49.9–51.1

Bivariate analysis at the individual level showed that women had better oral health than men (PR = 0.91, 95%CI 0.87–0.95) and the prevalence of bad oral health in non-white people was 40.0% higher than in white people (PR = 1.40, 95%CI 1.34–1.47). There was an increase in the prevalence of bad oral health as age increased (p < 0.001). The association with socioeconomic stratification and healthy eating habits also showed a dose-response effect. The worse the classification of the independent variable, the worse the prevalence of bad oral health.

A higher prevalence of bad oral health was associated to lower years of schooling expectancy (PR = 1.31, 95%CI 1.26–1.37), lower *per capita* income (PR = 1.45, 95%CI 1.39–1.52), higher income concentration (PR = 1.41, 95%CI 1.35–1.47), and worse human development (PR = 1.45, 95%CI 1.39–1.52). Inversely, oral health primary care services were negatively associated with oral health perception. A higher prevalence of good oral health was present in places with lower coverage for these services (Table 3).

**Table 3:** Bivariate associations between outcome and the independent variables according to the levels. Brazil, 2013.

Variable	Oral Health Self-Perception				p	PR (95%CI)

	Good/Very good		Regular to very bad			
	
	%	95%CI	%	95%CI		
1st level (individual)						

Sex						
Male	65.8	64.7–66.8	34.2	33.2–35.3	< 0.001	1
Female	68.9	68.0–69.8	31.1	30.2–31.9		0.91 (0.87–0.95)
Skin color						
White	73.1	72.2–74.1	26.9	25.9–27.8	< 0.001	1
Black or Mixed	62.3	61.3–63.3	37.7	36.7–38.7		1.40 (1.34–1.47)
Age (years)						
18 to 24	74.5	72.7–76.1	25.5	23.9–27.9	< 0.001	1
25 to 40	70.8	69.7–71.9	29.2	28.1–30.3		1.14 (1.06–1.24)
41 to 59	63.3	62.0–64.6	36.7	35.4–38.0		1.44 (1.34–1.55)
60 years and older	62.3	60.6–63.9	37.7	36.1–39.4		1.48 (1.36–1.60)
Literate						
Yes	68.9	68.2–69.6	31.1	30.4–31.8	< 0.001	1
No	51.6	49.2–54.1	48.4	45.9–50.8		1.55 (1.47–1.64)
Socioeconomic stratification						
A or B	74.9	73.5–76.3	25.1	23.7–26.5	< 0.001	1
C	67.4	66.4–68.5	32.6	31.5–33.6		1.30 (1.22–1.38)
D or E	61.8	60.7–62.8	38.2	37.2–39.3		1.53 (1.44–1.62)
Healthy eating habits						
Yes	72.5	71.5–73.4	27.5	26.6–28.5	< 0.001	1
No	61.8	60.8–62.8	38.2	37.2–39.2		1.39 (1.33–1.45)

2nd level (state)						

Years of Schooling Expectancy at 18 years old						
9.4 and more	71.0	70.0–72.0	29.0	28.0–30.0	< 0.001	1
Up to 9.4	61.9	60.9–62.9	38.1	37.1–39.1		1.31 (1.26–1.37)
*Per capita* income (BR R$)						
671 and more	71.8	70.8–72.7	28.2	27.3–29.2	< 0.001	1
Up to 670	59.0	58.0–60.0	41.0	40.0–42.0		1.45 (1.39–1.52)
Gini Index						
Up to 0.600	71.2	70.2–72.1	28.8	27.9–29.8	< 0.001	1
0.601 and more	59.5	58.4–60.5	40.5	39.5–41.6		1.41 (1.35–1.47)
Human Development Index (HDI)						
0.709 and more	71.8	70.8–72.7	28.2	27.3–29.2	< 0.001	1
Up to 0.708	59.0	58.0–60.0	41.0	40.0–42.0		1.45 (1.39–1.52)
Oral Health Primary Care						
14.7 and more	63.8	62.8–64.9	36.2	35.1–37.2	< 0.001	1
Up to 14.6	71.0	69.9–72.0	29.0	28.0–30.1		0.80 (0.77–0.84)

Concerning multilevel modeling, the initial null model indicated that there is compelling evidence that the between-community (in our case, states) variance is non-zero, as the Likelihood Ratio (LR) test statistic is 502.05 with a corresponding p-value ≤ 0.001 (Table 4). In model 1, when only the individual variables were included, all of them remained significant. The PR values and their respective confidence intervals were slightly adjusted in relation to bivariate analysis. When the contextual variables were included, a collinear effect between *per capita* income and HDI was observed, so the first one was not included in the next model. In the final model, variables that lost statistical significance were excluded; however, we included the last block with variable related to oral health services. The final model consisted of all individual variables except “literacy” and the contextual variables “HDI” and “oral health primary care”, which remained significant. All estimates were adjusted at the final model and the LR test showed that the contextual effect maintained its significance. A better adjustment was verified for HDI and socioeconomic stratification (especially “D or E” class).

**Table 4:** Poisson multilevel regression analysis for bad oral health self-perception according to individual and contextual variables. Brazil, 2013.

Variable	Null Model (n = 60,202)	Model 1 (n = 60,199)		Model 2 (n = 60,199)		Final Model (n = 60,199)	

		PR (95%CI)	p	PR (95%CI)	p	PR (95%CI)	p
1st Level (individual)							

Sex							
Male		1		1		1	
Female		0.94 (0.92–0.97)	< 0.001	0.94 (0.92–0.97)	< 0.001	0.94 (0.91–0.97)	< 0.001
Skin color							
White		1		1		1	
Black or Mixed		1.19 (1.15–1.23)	< 0.001	1.18(1.15–1.22)	< 0.001	1.19(1.16–1.23)	< 0.001
Age (years)							
18 to 24		1		1		1	
25 to 40		1.16 (1.10–1.22)	< 0.001	1.16(1.10–1.22)	< 0.001	1.17(1.11–1.22)	< 0.001
41 to 59		1.44 (1.37–1.51)	< 0.001	1.44 (1.37–1.51)	< 0.001	1.47 (1.40–1.54)	< 0.001
60 and older		1.49 (1.42–1.58)	< 0.001	1.50 (1.42–1.58)	< 0.001	1.57 (1.49–1.65)	< 0.001
Literate							
Yes		1		1		–	
No		1.17 (1.12–1.22)	< 0.001	1.17 (1.12–1.22)	< 0.001	–	
Socioeconomic stratification							
A or B		1		1		1	
C		1.18 (1.13–1.23)	< 0.001	1.17 (1.13–1.22)	< 0.001	1.18 (1.13–1.23)	< 0.001
D or E		1.24 (1.19–1.29)	< 0.001	1.24 (1.17–1.29)	< 0.001	1.27 (1.21–1.31)	< 0.001
Healthy eating habits							
Yes		1		1		1	
No		1.32 (1.28–1.36)	< 0.001	1.31 (1.27–1.35)	< 0.001	1.33 (1.29–1.37)	< 0.001

2nd level (state)							

Years of Schooling Expectancy at 18 years old							
9.4 and more				1		-	
Up to 9.4				1.02 (0.95–1.10)	0.503	-	
Gini Index							
Up to 0.600				1		-	
0.601 and more				1.02 (0.94–1.10)	0.659	-	
Human Development Index (HDI)							
0.709 and more				1		1	
Up to 0.708				1.16 (1.07–1.26)	< 0.001	1.19 (1.12–1.26)	< 0.001
Oral Health Primary Care							
14.7 and more						1	
Up to 14.6						0.93 (0.88–0.98)	0.009
Fixed Effects							
Intercept (95%IC)	-1.06 (-1.13– -0.99)	0.18 (0.17–0.19)		0.16 (0.15–0.17)		0.16 (0.15–0.18)	
Random effects							
Variance (95%CI)	0.029 (0.016–0.050)	0.011 (0.006–0.021)		0.004 (0.002–0.008)		0.003 (0.002–0.007)	
LR test (x^2^, p-value)	502.05 (< 0.001)	143.03 (< 0.001)		30.07 (< 0.001)		18.08 (< 0.001)	

LR: Likelihood Ratio

There was a steady decline in the values of variance from the null model to the final, starting with 62.0% from null model to the first and ending with an overall reduction of 90.0%, showing a strong effect of the context, considering the state federative units in Brazil. The inclusion of the interaction term, created from socioeconomic stratification and HDI, did not cause a significant modification in the variance in the adjusted final model. To confirm this assumption, we also performed stratified analyses, observing the values of RP for the association between oral health perception and socioeconomic stratification for each category of HDI. There were no differences in these values, therefore, these results indicate that there are no cross-level interactions.

## DISCUSSION

Both individual factors and characteristics of the context where people live were synergistically associated in their perception of oral health, as has been found in other studies^11^ ^,^ ^14^ ^,^ ^20^. This study has proposed a multilevel analysis to assess the social determinants of self-reported oral health status, understanding that only individual factors cannot be sufficient to explain how oral health status is perceived by people. Our perspective is that an individual is embedded in a collective context with social, economic, and political characteristics, which profoundly differ regardless of the scale of territory, as inequalities can be expressed in countries, states, cities and even neighborhoods.

Human Development Index, as a measure of the quality of life, maintained a significant effect, thus showing that better oral health is present in individuals living in places with higher HDI. This fact shows us the importance of investment in social public policies to enhance the population’s quality of life, and hence their oral health perception^21^. Other contextual variables, such as Gini index, lost their significance after adjustment, very likely due to its small variation among the Brazilian federative units. According to Barros et al.^22^, despite showing better indicators in the South and Southeast regions, income inequality is still uniform throughout the whole country, probably because inequality is affected more by national economic policies and less by state-level interventions. Wilkinson and Pickett^23^ pointed out that the association between income inequality and health are more likely to be found in studies performed with large areas, usually international studies that use countries as a unit of analyses. In studies where the analyses were done in states, cities or regions, even in small units, such as counties, neighborhoods or census tracks, the results are usually inconclusive.

The results for oral health services in primary care have shown that places with higher coverage for these services also have a higher prevalence of bad self-perceived oral health. Although this could be counterintuitive, it can be interpreted that there has been a more equitable distribution of public resources because a major amount of them have been deployed to the neediest places. Working with cross-sectional data (as we did), it is impossible to assess whether public health services would have a mitigating effect on the inequalities in oral health; such an effect could only be assessed through longitudinal studies. However, our results are in accordance with the current principles of the Brazilian National Oral Health Policy in Brazil (PNSB), which states that the distribution of resources must be based on equity^24^.

Nevertheless, in analyzing the implementation of the PNSB, it is possible to notice after more than a decade that the most vulnerable social regions where these health services have been deployed are still showing worse indicators. This means that, actually, there was no significant change in the oral health assistance model^25–27^.

Secondary oral health services have lost their significance in the adjusted model. According to Soares^28^, despite the quantitative expansion of oral health specialized services in the last decade in Brazil, difficulties in accessing these services persist in many regions. Moreover, Góes et al.^29^ found that the low coverage of oral health in primary care alongside its inadequate structure are both important factors that compromise comprehensive specialized oral health care.

Regarding the determinants of the work and life conditions, several studies have shown that educational status is an important factor in the oral health determination^1^ ^,^ ^5^ ^,^ ^11^ ^,^ ^20^ ^,^ ^22^ ^,^ ^30^, which was not found in our study. This is probably because the variable “literacy” has poor discriminant power, as can be confirmed by the high number of literate people in the whole sample. On the other hand, socioeconomic stratification also showed a dose-response effect, even after adjustment, i.e., the lower the socioeconomic status, the worse the oral health condition. Similar findings were observed by other authors^1^ ^,^ ^5^ ^,^ ^14^ ^,^ ^20^. Regarding household crowding, this variable reflects the family economic situation, as the more people living in small houses, the less the family income. Thus, economically privileged families (as measured in this study) essentially represent the families’ purchasing power, so it is expected that these family members have more access to health services, more information about oral health prevention, and consequently, have better oral health.

Individual behaviors, personal choices, and lifestyle also showed an important role in oral health perception determination, as was also found by Gabardo et al.^14^ In our study, people with healthy eating habits and those that related using dental floss presented better oral health, even after the adjustment for all other variables, which also has strong scientific evidence^14^ ^,^ ^31^. Although choosing a healthy lifestyle could be considered as an individual decision, it is also socially influenced through economic and cultural issues alongside access to health services; therefore, it is considered a social determinant^21^.

We found better oral health in women compared to men, which disagrees with the results of some authors^1^ ^,^ ^14^. However, our findings could be explained by the fact that women usually have more focused attitudes on oral health care compared to men^1^ and hence express better oral health perception^32^. In relation to age, we observe a dose-response effect, even after adjustment, i.e., the higher the age the worse is the oral health condition. Dental caries and periodontal diseases usually have a cumulative effect, so older people have a life trajectory of suffering because of these diseases, and thus report bad oral health more often. Similar findings were found by several authors^1^ ^,^ ^14^ ^,^ ^20^. In relation to skin color, worse oral health was referred by non-white people, in accordance with Barbosa et al.^33^ Such an outcome was expected, as in most studies in Brazil, race (or ethnicity, skin color) can be considered as a proxy of socioeconomic status^34^ ^,^ ^35^, but it is noteworthy that race maintained its significance even in the presence of other variables such as socioeconomic stratification. This could indicate that skin color is in itself an important marker in oral health inequalities^36^.

Some studies using multilevel techniques have found a strong relationship between social disparities and health inequalities^25,37–39^. Multilevel modeling has been adopted in several studies as a powerful instrument to elucidate the influence of both individual and contextual factors on health population. Nevertheless, it is also important to understand the subjectivity involved in oral illness and how these interlaced characteristics could reflect on oral health perception, which is affected by indicators beyond those related to clinical conditions. In order to promote better oral health to the population, it is crucial to include measures that tackle both the main individual factors and those related to quality of life and egalitarian social policies.

In this study, the aggregation unit used was the Brazilian federation unit, due to the availability of representative data in this level of aggregation. However, this unit of aggregation is not ideal because, within it, there is still a lot of context variability. Our explanatory theoretical model is still reductionist in face of the complexity of factors that affect the social context regarding individuals and their perception. However, such limitations do not invalidate the findings here. Further studies may complement these findings and deepen the discussion here.

The bad oral health perception is determined by a combination of biological, proximal, and distal factors, as discussed in our framework. These gradients of oral health related to these factors can be considered unfair and avoidable, which allude to social inequalities.
